# Mutations in *B9D1* and *MKS1* cause mild Joubert syndrome: expanding the genetic overlap with the lethal ciliopathy Meckel syndrome

**DOI:** 10.1186/1750-1172-9-72

**Published:** 2014-05-05

**Authors:** Marta Romani, Alessia Micalizzi, Ichraf Kraoua, Maria Teresa Dotti, Mara Cavallin, László Sztriha, Rosario Ruta, Francesca Mancini, Tommaso Mazza, Stefano Castellana, Benrhouma Hanene, Maria Alessandra Carluccio, Francesca Darra, Adrienn Máté, Alíz Zimmermann, Neziha Gouider-Khouja, Enza Maria Valente

**Affiliations:** 1 IRCCS Casa Sollievo della Sofferenza, Mendel Laboratory, Neurogenetics Unit, San Giovanni Rotondo, Italy; 2 Department of Medical and Surgical Pediatric Sciences, University of Messina, Messina, Italy; 3 Research Unit 06/11 and Department of Child and Adolescent Neurology, National Institute Mongi Ben Hmida of Neurology, Tunis, Tunisia; 4 Department of Medical, Surgical and Neurological Sciences, University of Siena, Siena, Italy; 5 Unit of Child Neuropsychiatry, Policlinico G.B. Rossi, Verona, Italy; 6 Department of Paediatrics, Faculty of Medicine, University of Szeged, Szeged, Hungary; 7 Department of Neurosurgery, Faculty of Medicine, University of Szeged, Szeged, Hungary; 8 Department of Medicine and Surgery, University of Salerno, Salerno, Italy

**Keywords:** Joubert syndrome, Meckel syndrome, Ciliopathies, Primary cilium, *MKS1*, *B9D1*, Genotype-phenotype correlates

## Abstract

Joubert syndrome is a clinically and genetically heterogeneous ciliopathy characterized by a typical cerebellar and brainstem malformation (the “molar tooth sign”), and variable multiorgan involvement. To date, 24 genes have been found mutated in Joubert syndrome, of which 13 also cause Meckel syndrome, a lethal ciliopathy with kidney, liver and skeletal involvement. Here we describe four patients with mild Joubert phenotypes who carry pathogenic mutations in either *MKS1* or *B9D1*, two genes previously implicated only in Meckel syndrome.

## Findings

### Background

Joubert syndrome (JS, MIM213300) is a congenital disorder diagnosed by the presence of a peculiar midbrain-hindbrain malformation (the “molar tooth sign”, MTS), that consists of cerebellar vermian hypodysplasia, thickened mal-oriented superior cerebellar peduncles, and a deepened interpeduncular fossa. The typical neurological features of pure JS include hypotonia, ataxia, psychomotor delay, abnormal ocular movements, intellectual impairment of variable degree, and often breathing abnormalities. This phenotype may be complicated by defects of the kidneys (nephronophthisis), eyes (retinal dystrophy or colobomas), liver (congenital fibrosis), skeleton (mainly polydactyly), and orofacial defects (cleft lip and/or palate, tongue hamartomas), resulting in wide clinical variability [1].

JS is recessively inherited and genetically heterogeneous, with 24 known genes that overall account for about half cases. All genes encode for proteins of the primary cilium, and indeed there is clinical and genetic overlap with other ciliopathies. In particular, JS shares 13 genes with Meckel syndrome (MS, MIM249000), a lethal condition characterized by cystic kidneys, bile duct proliferation of the liver, encephalocele and polydactyly. Other malformations frequently include cleft lip and palate, bowing of long bones and other skeletal defects, and situs inversus [2].

### Identification of *MKS1* and *B9D1* mutations in JS patients

As part of a large screening of ciliopathy genes in 260 JS patients, we identified novel pathogenic mutations in two genes not previously implicated in this condition.

**Figure 1:**
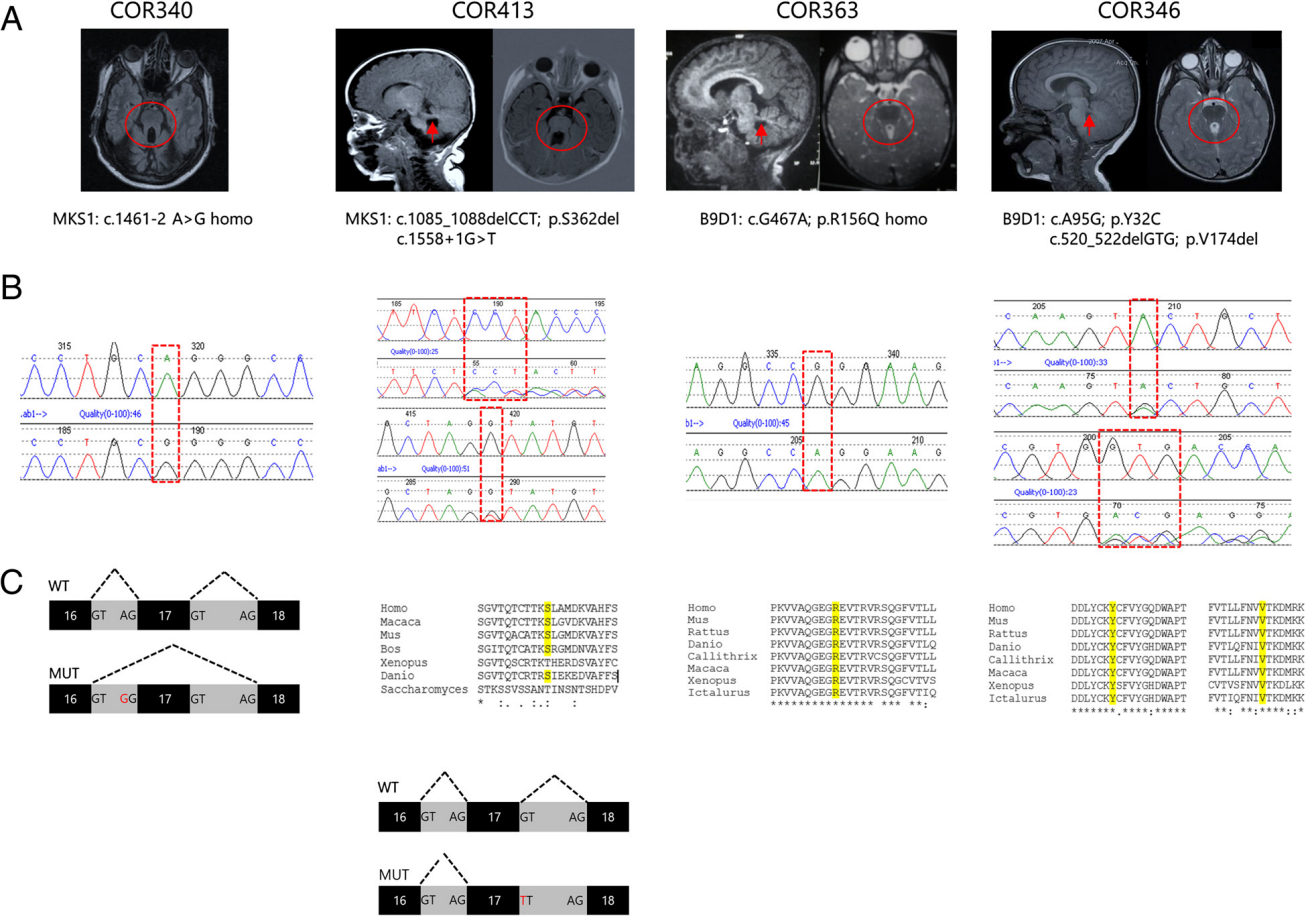
**Brain magnetic resonance imaging and characterization of mutations of the four patients. A**) Parasagittal (left) and axial (right) magnetic resonance imaging sections showing thickened and elongated superior cerebellar peduncles (arrows), and the molar tooth sign (circles). Parasagittal images are not available for patient COR340. **B**) Electropherograms showing the identified mutations; **C**) conservation of affected amino acid residues among orthologues (for missense mutations or single amino acid deletions), or prediction of aberrant splicing (for splice-site mutations).

Two patients carried mutations in the *MKS1* gene [GenBank:NG_013032.1], a 44-year-old man with JS and retinal dystrophy (COR340), and a 2-year-old child with a pure JS phenotype (COR413). Mutations in the *B9D1* gene [GenBank:NG_031885.1] were identified in two other patients, a 9-year-old boy (COR363) and a 7-year-old girl (COR346), both presenting with pure JS. All identified mutations were inherited from heterozygous healthy parents, were not reported in public databases, and affected highly conserved residues (Figure 1). Missense mutations were predicted as pathogenic by prediction web tools. Clinical features of the four patients, compared with the phenotypes of the six JS subgroups [1], are summarized in Table 1. Individual case reports and details on genetic analysis are described in the Additional file 1.

**Table 1:** Phenotypic comparison of the four patients presented here with JS clinical subgroups

	**Present cases**				**JS clinical subgroups (as in ref 1)**					
	**COR340 ( *MKS1 *)**	**COR413 ( *MKS1 *)**	**COR363 ( *B9D1 *)**	**COR346 ( *B9D1 *)**	**Pure**	**With retina**	**With kidney**	**With retina & kidney**	**With liver**	**OFD-VI**
**CNS:**										
- hypotonia/ataxia	+	+	+	+	+	+	+	+	+	+
- breathing abn.	-	-	-	-	±	±	±	±	±	±
- develop. delay	+	+	+	+	+	+	+	+	+	+
- ID	+	+	+	-	±	+	+	+	+	±
- oculomotor abn.*	+	+	+	+	±	±	±	±	±	±
**Ocular:**										
- retinopathy	+	-	-	-	-	+	-	+	-	-
- coloboma	-	-	-	-	±	rare	rare	rare	±	rare
**Renal:**	-	-	-	-	-	-	+	+	±	rare
**Hepatic:**	-	-	-	-	-	-	-	rare	+	-
**Other features:**										
- polydactyly	-	-	-	-	±	rare	rare	rare	rare	±
- orofacial features	-	-	-	-	-	-	-	-	-	±
- dysmorphisms	+	-	+	+	±	±	±	±	±	±
**Neuroimaging:**										
- MTS	+	+	+	+	+	+	+	+	+	+
- other CNS defects**	-	-	-	-	rare	rare	rare	rare	rare	±

Legend: For the six JS subgroups, the meaning of symbols is as follows: “+”: mandatory feature; “±”: feature that could be part of the phenotype but is not mandatory; “rare”: feature that was only rarely described in the subgroup; “-“: never described to date. *mainly oculomotor apraxia and/or nistagmus; **mostly including corpus callosum hypoplasia, encephalocele, neuronal migration defects (e.g. polymicrogyria), hypothalamic hamartoma (in OFD-VI).

Abbreviations: *abn* abnormalities, *CNS* central nervous system, *develop* developmental, *ID* intellectual disability of variable severity, *MTS* molar tooth sign.

## Discussion

Pathogenic mutations in *MKS1* and *B9D1* have been reported only in MS fetuses. *MKS1* is mutated in about 7-14% of MS patients, with increased frequency in northern European countries due to a founder mutation [3-7]. Several studies have highlighted that mutations in *MKS1* are associated with a particularly severe MS phenotype, with high occurrence of polydactyly, bone dysplasia, encephalocele and other central nervous system anomalies [4-6]. To date, *B9D1* was found mutated only in one MS fetus with cystic dysplastic kidneys, encephalocele, shortened limbs and ambiguous genitalia [8]. Conversely, the four JS patients described here all had a relatively mild presentation characterized by a pure neurological phenotype, with the exception of retinal dystrophy in patient COR340. The degree of intellectual impairment was variable, and patient COR413 even presented with normal intellectual abilities, a rare occurrence in JS [9]. None of the patients showed involvement of the organs that are typically affected in MS, namely the kidneys, liver and skeleton, although a future renal disease can be safely excluded only in the adult patient (COR340).

This wide phenotypic variability associated with mutations in the same genes remains an intriguing open question. Genotype-phenotype correlates have been proposed for some genes (such as *RPGRIP1L*, *TMEM67*, *CCD2D2A* and *TCTN3*), with biallelic null mutations causative of lethal phenotypes, and at least one hypomorphic missense mutation found in JS [10-13]. This could also hold true for *MKS1* and *B9D1*. In fact, most MS fetuses are known to carry two null mutations in these genes [4,8]; conversely, three of our JS patients have at least one mutation not resulting in protein truncation, and the fourth is homozygous for a splice-site mutation involving the penultimate exon of *MKS1*, whose pathogenetic impact on the protein remains to be determined (See Additional file 1) (Figure 1). Interestingly, a previous study identified two hypomorphic mutations in the *MKS1* gene (a missense change and a single aminoacid deletion) in a 2-year-old Turkish patient with Bardet-Biedl syndrome, another non-lethal ciliopathy partly overlapping with JS, supporting this hypothesis [14]. Yet, these genotype-phenotype correlates are unlikely to fully explain the extreme phenotypic variability of these allelic ciliopathies, and other mechanisms, such as the presence of modifier variants in other genes, need to be explored.

MKS1, B9D1 and B9D2 proteins are known to interact physically [15], and are main components of the “B9” or “Tectonic” complex residing at the ciliary transition zone, that includes many other proteins mutated in JS and/or MS [16]. In our large JS cohort, *MKS1* and *B9D1* mutations each account for less than 1% cases. We failed to identify mutations in *B9D2*, but we cannot exclude that this gene may also be rarely mutated in JS.

In conclusion, we expand the genetic basis of JS to include *MKS1* and *B9D1*, delineate genotype-phenotype correlates, and further outline JS and MS as the two ends of a common spectrum. These findings have implications for genetic testing and counselling of JS patients and their families.

## Abbreviations

JS: Joubert syndrome; MS: Meckel syndrome; MTS: Molar tooth sign.

## Competing interests

The authors declare that they have no competing interests.

## Authors’ contributions

Patients’ recruitment, data collection, analysis of clinical and imaging data: IK, MTD, MC, LS, FM, BH, MAC, FD, AM, AZ, NGK, EMV; molecular genetic studies: MR, AM, RR; bioinformatics analysis: TM, SC; study conception and design, manuscript drafting: MR, EMV. All authors revised the manuscript critically and approved the final version.

## Supplementary Material

Additional file 1 **Supplementary material.** Supplementary Methods. Prediction of the effect of *MKS1* splice-site mutations. Case Reports. Click here for file
