# Supplementary material for: Mutations in B9D1 and MKS1 cause mild Joubert syndrome: expanding the genetic overlap with the lethal ciliopathy Meckel syndrome
Source: Orphanet J Rare Dis. 2014 May 5;9:72. doi: 10.1186/1750-1172-9-72 (PMC4113192; doi:10.1186/1750-1172-9-72)
Supplement: Additional file 1 — Supplementary material. Supplementary Methods. Prediction of the effect of MKS1 splice-site mutations. Case Reports. [file 1750-1172-9-72-S1.pdf]

Supplementary material

Supplementary methods

Simultaneous target sequencing of 50 ciliopathy genes (including most genes known to cause human primary ciliopathies) was performed on a Solid 5500xL platform (Life Technologies) in a total of 260 probands representative of the whole clinical spectrum of JS, recruited over the past ten years at the IRCCS CSS-Mendel Institute in Rome, Italy. All patients had neuroradiologically proven MTS. For each patient, a detailed clinical questionnaire filled by the referring clinician allowed to obtain information on the extent of organ involvement. In particular, nearly all patients underwent measurement of renal and hepatic function, abdominal ultrasound, assessment of visual ability and fundoscopy. Written informed consent was obtained from all families, and the study was approved by the local ethics committee.

The identified mutations in *MKS1* and *B9D1* were verified using bidirectional Sanger sequencing, and were searched against public databases dbSNP ver.138 (http://www.ncbi.nlm.nih.gov/SNP/) and Exome Variant Server (http://evs.gs.washington.edu/EVS/). The potential pathogenicity of the two *B9D1* missense mutations (p.R156Q and p.Y32C) was predicted using PolyPhen-2 ver.2.2.2 (http://genetics.bwh.harvard.edu/pph2/), SIFT (http://sift.jcvi.org/) and Mutation Assessor (http://www.mutationassessor.org) web tools. Clustal Omega (http://www.ebi.ac.uk/Tools/msa/clustalo/) was employed to assess evolutionary conservation of mutated amino acid residues by multiple sequence alignment of human MKS1 and B9D1 proteins (NP_001159399.1 and NP_056496.1) and their orthologues. To

predict the effect of the two *MKS1* splice-site mutations (c.1461-2A>G and c.1588+1G>T) on the resulting proteins, we used Variant Effect Predictor software (http://www.ensembl.org/info/docs/tools/vep). Mutation nomenclature was assigned according to the Human Genome Variant Society (http://www.hgvs.org/mutnomen/).

Prediction of the effect of *MKS1* splice-site mutations

The Variant Effect Predictor software predicted *MKS1* c.1461-2A>G to be a splice acceptor variant that would lead to skipping of exon 17. This would result in a frameshift with substitution of the last 62 amino acids of the protein with 59 different amino acids before protein truncation (p.A488Gfs*59). Conversely, *MKS1* c.1588+1G>T was considered a splice donor variant, causing retention of intron 17. In turn, this would generate a protein of the same length as the wild type, in which the last 30 amino acids are modified (p.E520Gfs*30). Unfortunately, fibroblasts from these two patients were not available to conduct mRNA studies, and the possibility that either of these two splice-site mutations may result in nonsense-mediated RNA decay cannot be formally excluded.

Case reports

COR340 (MKS1 c.1461-2 A>G homozygous)

This 44-year-old man was born two weeks preterm (weight at birth 2900g) after an uneventful pregnancy from unrelated Italian parents. Unspecified severe visual problems were referred in the paternal grandmother and two other relatives. Since neonatal age, the patient presented hypotonia, strabismus, abnormal eye movements and developmental

delay, and was able to walk unaided with ataxic broad-based gait at age 5 years. At age 2 years, a diagnosis of pigmentary retinopathy was made, but no further details are available. He attended school up to the age of 14 years with poor profit. He was first examined by a neurologist (MTD) at age 15 years. Clinical examination showed facial dysmorphisms including broad forehead, wide-spaced eyes, arched eyebrows and broad nasal bridge, flat valgus feet, slim and elongated hands with marked ligamentous laxity. Neurological examination revealed gait ataxia, poor vision, strabismus, nystagmus, dysarthria with slurred speech, mild dysmetria, and hyperactive tendon reflexes especially in the lower limbs. There was mild intellectual impairment, with an IQ of 68. Fundoscopy confirmed the diagnosis of pigmentary retinopathy, with pale optic discs, while electroretinography was not recordable. Brain MRI detected the MTS, allowing to diagnose JS. Subsequently, the patient was periodically re-evaluated up to the present age of 44 years. No significant changes were observed over time, except the occurrence of bilateral ptosis and ginecomastia not associated with hyperprolactinemia, since the age of about 20 years.

COR413 (MKS1: c.1085_1088delCCT; p.S362del + c.1558+1G>T)

This 2-year-old boy was born from non-consanguineous parents originating from Romania and Moldavia, respectively. Fetal ultrasound at 24 gestational weeks showed hypoplasia of the cerebellar vermis and an enlarged fourth ventricle. Auxological parameters at birth were normal, with APGAR scores of $9^{1'}$ and $10^{5'}$. The patient presented early hypotonia, oculomotor apraxia, and marked psychomotor delay. A brain MRI, performed at 5 months, showed the “molar toot sign” (MTS) leading to the diagnosis of Joubert Syndrome. The latest examination at age 2 years revealed axial hypotonia, mild limb hypertonia and ataxia

with intentional tremor and dysmetria. He was able to sit with support for a short time, and could speak a few syllables. There was severe intellectual impairment, with Griffiths general quotient (GQ) of 29. Fundus oculi, abdominal ultrasound, renal and hepatic function were all normal.

COR363 (B9D1: c.G467A; p.R156Q homozygous)

This 9-year-old Tunisian boy was born from third-degree consanguineous parents after a pregnancy complicated by gestational diabetes. Birth weight was high (4250gr), APGAR scores were $9^{1'}$ and $10^{5'}$. In the neonatal age, the patient had hypoglycemia. He presented with hypotonia, psychomotor delay, oculomotor apraxia and right convergent strabismus. Facial dysmorphisms included a triangular face, retrognatism, accentuated philtrum and big ears. Diagnosis of JS was made at age 1 year, upon identification of the MTS on brain MRI. Fundus oculi examination and electroretinogram, performed at age 3 years, were normal, with the only mention of possibly narrow retinal arteries. Hepatic and renal functions were repeatedly normal, while abdominal ultrasound showed bilateral pyelic ectasia in the absence of other malformations of the explored organs. At the latest follow-up at age 5 years, the boy presented mild intellectual impairment, axial hypotonia and gait ataxia.

COR346 (B9D1: c.A95G; p.Y32C + c.520_522delGTG; p.V174del)

This 7-year-old girl was born from Hungarian unrelated parents. Pregnancy and delivery were unremarkable. Oculomotor apraxia and delayed psychomotor skills were noted since the first months of life. In particular, she showed a significant language delay mainly in its

expressive component, but achieved a fluent language at age 5 years. Dysmorphic facial features included frontal bossing, macrostomia, thick lips and low-set ears. Neurological examinations performed at 4 and then at 6 years showed ataxia with altered coordination of gross and fine movements and oculomotor apraxia. However, interestingly, intellectual abilities tested with the Binet-Simon test resulted above average (IQ 118 at age 4.5 years). Hepatic and renal function and abdominal ultrasound were all normal, and she had no other associated malformations. Brain MRI was performed at age 3 years showing the MTS.
